# Efficacy and safety of left bundle branch area pacing performed with stylet-driven leads

**DOI:** 10.3389/fcvm.2026.1912017

**Published:** 2026-08-21

**Authors:** Lin Liang, Liping Li, Tong Wei, Xiaofeng Lu, Yan Liu, Anmin Ren, Weijie Zhu, Jun Li, Shaowen Liu, Songwen Chen, Juan Xu

**Affiliations:** Department of Cardiology, Shanghai General Hospital, Shanghai Jiao Tong University, School of Medicine, Shanghai, China

**Keywords:** conduction system pacing, left bundle branch area pacing, physiological pacing, site-selective pacing catheter (SSPC), Stylet-driven lead

## Abstract

**Background:**

Left bundle branch area pacing (LBBAP) has emerged as a physiological pacing strategy. LBBAP has been predominantly performed using the lumen-less lead.

**Objectives:**

This study aims to explore the efficacy and safety of LBBAP performed with stylet-driven leads (SDL) via site-selective pacing catheter (SSPC) sheaths.

**Methods:**

We evaluated consecutive 79 patients undergoing LBBAP using standard stylet-driven active leads via SSPC sheaths. Pacing parameters (threshold, R-wave amplitude, impedance), QRS morphology and duration, left ventricular activation time in lead V5 and complication rates were analyzed. Postoperative measurements of lead parameters and complications were recorded at 1, 3, 6, and 12 months.

**Results:**

LBBAP was successfully achieved in 69 of the 79 patients (87.3%), with 7742 leads via SSPC sheaths, with 52 patients (65.8%) showing visible left bundle branch potentials. Mean paced QRS duration and left ventricular activation time in lead V5 were 111.1 ± 27.2 ms and 69.0 ± 11.7 ms. Intraoperative electrode parameters for SDL-LBBAP, including pacing threshold (0.8 ± 0.2 V at 0.4 ms), impedance (792.5 ± 140.7 ohms), and sensing amplitude (14.0 ± 5.9 mV), remained stable during the 12 month follow up. Intraoperative ventricular septal perforation occurred in six patients (7.6%), with no subsequent persistent ventricular septal defects. Procedural time and fluoroscopy exposure time were lower in the latter 39 patients, declining markedly as operator experience accumulated.

**Conclusions:**

The application of SDL in LBBAP is safe and feasible, with excellent success rates and stable long term electrical performance.

## 1 Introduction

Implantation of cardiac pacemakers is an important method for treating bradycardia. Conventional right ventricular pacing (RVP) may lead to dyssynchronous cardiac contraction, pacing-induced cardiomyopathy, and even increased mortality. Conduction system pacing (CSP) has emerged as an alternative approach to achieve physiological pacing, primarily including His bundle pacing (HBP) and left bundle branch area pacing (LBBAP). HBP is considered the most physiological (1), but it is limited by high pacing thresholds, low sensing amplitude, and lower implantation success rates in patients with infra-nodal conduction abnormalities. LBBAP has become an alternative to physiological pacing as it provides hemodynamic effects comparable to HBP but with lower and more stable pacing thresholds, higher sensing amplitudes, and higher implantation success rates (2–4).

Most conduction system pacing currently uses the lumenless leads (LLLs, 3830, Medtronic, Inc.) and C315 sheath (Medtronic, Inc.). While most published studies have used LLLs, there is increasing interest in implanting stylet-driven leads (SDLs), given the wider general experience and availability of these leads across manufacturers (1, 5, 6).

Compared with LLLs, SDLs offer several practical advantages, including greater stiffness facilitating septal penetration, shorter procedural and fluoroscopic times, and the ability to continuously monitor QRS morphology during lead positioning. Importantly, SDLs achieve comparable success rates for left bundle branch capture.

Despite these benefits, data on the long-term efficacy and safety of LBBAP performed with SDLs is lacking, particularly for the 7742 lead (Boston Scientific, Marlborough, MA) deployed via the site-selective pacing catheter (SSPC, CenterPoint Systems West Valley City, UT) delivery sheath. In this study, we report the efficacy and safety of the SDL-LBBAP via the SSPC delivery sheath.

## 2 Materials and methods

### 2.1 Patients and devices

All patients provided written informed consent, who underwent a LBBAP attempt for a bradycardia indication. All LBBAP implant attempts using 7742 leads via SSPC sheaths were collected. The 7742 lead is a 6.0 Fr active fixation, SDL with an electrically active helix. The SSPC sheaths include 4 fixed-curve sheaths. The sheath was selected according to preoperative ultrasonic measurements of right atrial size and vascular access route. Patients were required to have a 12-month follow-up.

### 2.2 Procedural steps

Before the start of the case, echocardiography was reviewed to assess the interventricular septal (IVS) thickness. Via the axillary or subclavian venous approach, a 9Fr sheath was inserted. A device pocket was also created at the plane of the pectoralis muscle. Under fluoroscopy in a right anterior oblique (RAO) 30° view, the SSPC sheath was advanced across the tricuspid valve over a guidewire (0.035 inch, Ment Medical Systems, Inc). The wire was then removed, and the sheath remains in the main body of the RV. The preferred location is in the first third of the ventricle as visualized within the RAO view. Left anterior oblique (LAO) 40°and RAO 30° projections were employed to guide septal engagement and depth assessment. The sheath could also be reshaped to achieve better positioning on the ventricular septum when within the heart, for example, to lift the catheter higher on the septum. To obtain real-time unipolar electrograms from the lead tip, alligator clips were attached with the black clip to the stylet and the red clip to the subcutaneous pocket. The body of SDL and the torque wrench were rotated clockwise simultaneously. Ventricular pacing was delivered from the right side of the interventricular septum (IVS) at an output of 5.0 V and 0.4 ms. A left bundle branch block (LBBB) pattern of QRS complex in lead V1 indicated pacing at the right bundle branch or its adjacent region. The pacing lead was then gradually screwed toward the left side of the ventricular septum. To avoid retraction of the electrode helix during rotation, the lead body and torque wrench were rotated synchronously while closely monitoring impedance and R-wave amplitude. When a notch appeared at the terminal portion of the QRS complex, the QRS morphology and impedance were closely monitored. Subsequently, only the torque wrench was used to rotate the lead counterclockwise until a right bundle branch block (RBBB) morphology was achieved. In our practice, we routinely omitted contrast injection for lead depth verification, using ring pacing threshold, tip pacing morphology, and lead impedance to guide positioning, with no lead dislodgement noted thereafter. The sheath was withdrawn into the right atrium while maintaining appropriate bending tension at the lead tip under fluoroscopy, followed by sheath cutting and removal. If the lead position was unsatisfactory, the lead was rotated clockwise and extracted to check tissue entanglement and helix integrity, and the above implantation procedures were repeated.

### 2.3 Defining successful LBBAP

Successful LBBAP was defined as the achievement of selective-LBBAP （S-LBBAP）, non- selective LBBAP（NS-LBBAP）, or stable left ventricular septal pacing (LVSP) with satisfactory pacing parameters. The following criteria were used to define the pacing response (1, 2): (1) Unipolar pacing in lead V1 showed a right bundle branch block (RBBB) morphology (QR or rsR’); (2) The left ventricular activation time (LVAT) shortened significantly at high output, while remaining short and constant at low output. (3) A left bundle branch (LBB) potential was recorded; (4) An isoelectric line was present between the pacing spike and the onset of the QRS complex. Patients who fulfilled the 1-2 criteria with at least one other criterion were deemed to have LBBAP.

### 2.4 Data collection and follow-up

Intraoperative and postoperative follow-up measurements of pacing thresholds, sensing amplitudes, and lead impedance were systematically performed at predefined time points, including the intraoperative period and 1, 3, 6, and 12 months after pacemaker implantation. All electrophysiological parameters were measured from paced surface electrocardiograms and intracardiac signals recorded at the lead tip. Specifically, LVAT was defined as the interval from the pacing stimulus artifact to the peak of the R wave in lead V5 during pacing at an output of 5 V/0.4 ms. Additionally, procedural parameters, total procedure time and fluoroscopy exposure time were recorded. Total procedure time is defined as the skin-to-skin duration, including venous access, sheath preparation, lead implantation, device connection, and wound closure. In contrast, fluoroscopy time specifically represents the fluoroscopic exposure required during the LBBAP implantation procedure after the SSPC sheath crossed the tricuspid valve until successful ventricular lead fixation. Procedural complications were defined as cardiac perforation, tamponade, lead fracture, infection, thromboembolism, or pacing-induced cardiomyopathy.

### 2.5 Statistical analysis

Variables following a normal distribution were presented as Mean ± standard deviation (SD), while non-normally distributed variables were reported as median [interquartile range (IQR), 25th to 75th percentile]. Categorical variables were expressed as frequencies (percentages). Comparisons of characteristics were conducted using the Fisher's exact test for categorical variables and a Student t-test or Mann–Whitney U-test for continuous variables. All the analyses were performed with R software V.4.1.2. A two-sided *p* < 0.05 was considered statistically significant.

## 3 Results

**Figure 1:**
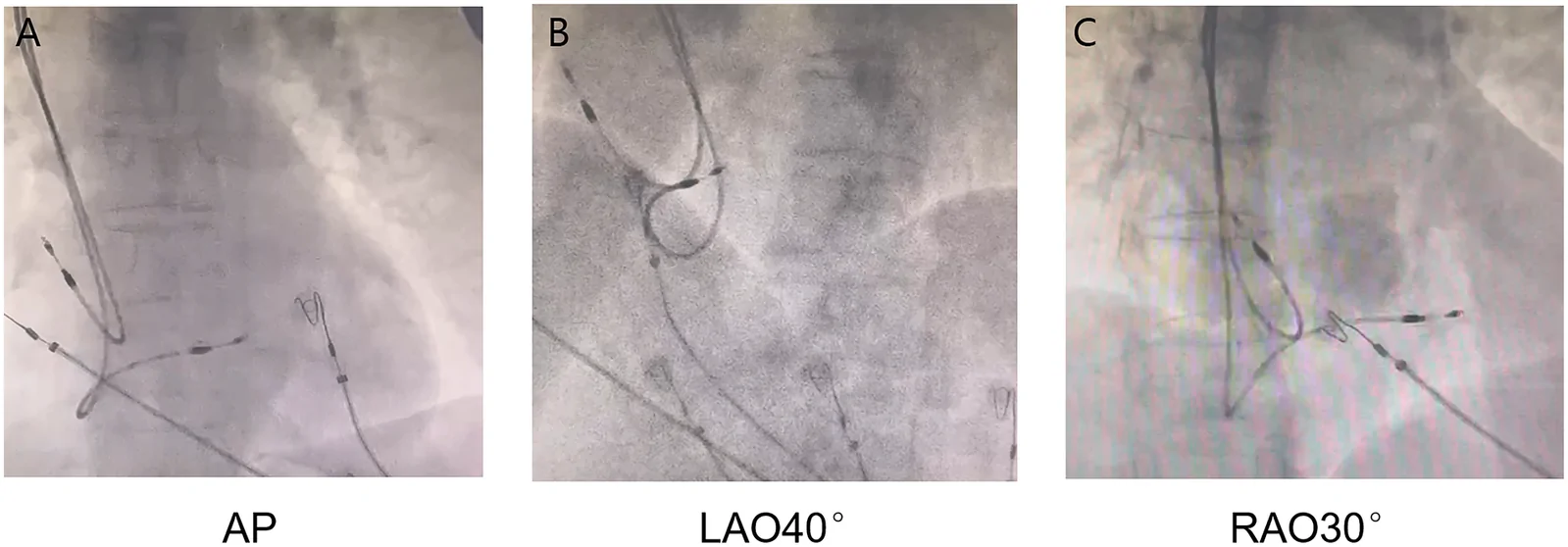
Fluoroscopy of leads. Representative x-ray images were acquired in anteroposterior **(A)**, left anterior oblique 40° **(B)**, and right anterior oblique 30°views **(C)**, respectively.

The SDLs were recorded in the anterioposterior (AP), steep LAO 40° and RAO 30° views (Figure 1). Baseline patient characteristics are described in Table 1. The average patient age was 74 years old, 50.6% were female, and the average left ventricular ejection fraction (LVEF) was 63.0 ± 3.8%. Common comorbidities included hypertension (22, 27.8%), diabetes (8, 10.1%), and coronary artery disease (3, 3.8%). Pacemaker indications were most commonly atrioventricular (AV) block (31, 39.2%), sinus node dysfunction (42, 53.2%) and atrial fibrillation with slow ventricular rate (6, 7.6%). The mean baseline unpaced QRS duration was 111.1 ± 27.2 ms.

**Table 1:** Baseline characteristics, procedural indications, and medical history.

Patient characteristics	*n* = 79
Age, years, mean ± SD	74.0[67.0, 80.0]
Female gender, *n* (%)	40 (50.6)
Male gender, *n* (%)	39 (49.4%)
Height，cm	161 ± 9.9
Weight，kg	59.0 ± 1.4
BMI, kg/m^2^	24.3 ± 4.6
LVEF^1^ (%)	63.0 ± 3.8
IVS, mm	9.0 ± 1.3
Baseline QRS duration, ms	111.1 ± 27.2
Procedure indications	
SSS^2^, *n* (%)	42 (53.2%)
AVB^3^, *n* (%)	31 (39.2%)
AF^4^ with slow ventricular rate, *n* (%)	6 (7.6%)
Medical history	
Coronary artery disease, *n* (%)	3 (3.8%)
Diabetes mellitus, *n* (%)	8 (10.1%)
Hypertension, *n* (%)	22 (27.8%）

1 LVEF, left ventricular ejection fraction.

2 SSS, sinus nodal disease.

3 AVB, atrioventricular block.

4 AF, atrial fibrillation.

**Figure 2:**
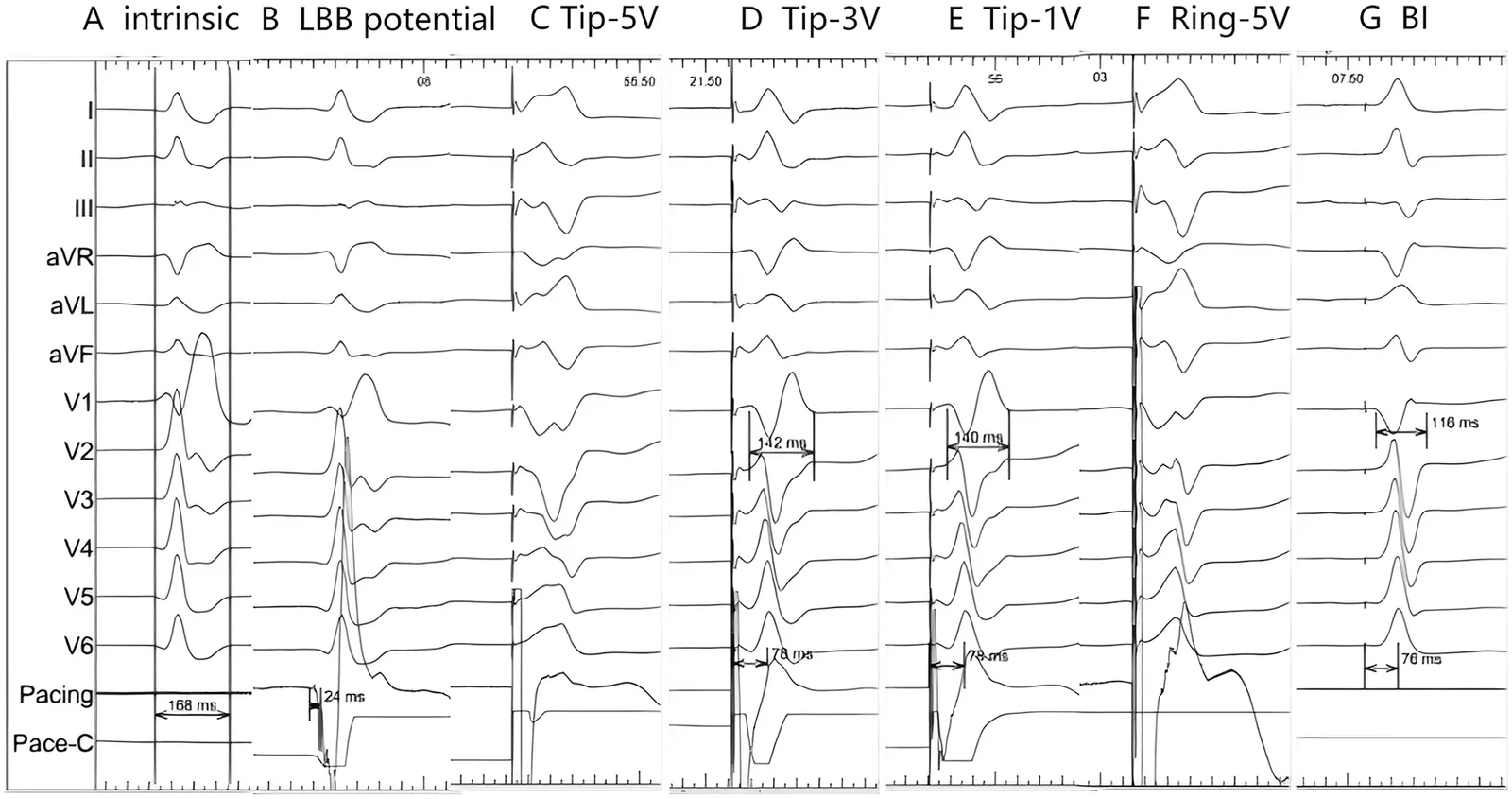
Electrocardiogram and potential recordings during lBBAP. **(A)** Preoperative ECG with the QRS duration of 168 ms. **(B)** Visible LBB potential with a P-V interval of 24 ms. **(C)** Tip pacing at 5 V showing a W-shaped waveform with the notch at the bottom of the QRS complex in lead V1. **(D)** Tip pacing at 3 V, presenting a qR pattern in lead V1; QRS duration 142 ms, LVAT 78 ms. **(E)** Tip pacing at 1 V, presenting a qR pattern in lead V1; QRS duration 142 ms, LVAT 78 ms. **(F)** Ventricular pacing via a ring electrode at 5 V. **(G)** Bipolar ventricular pacing; QRS duration 116 ms, LVAT 76 ms. LBB, left bundle branch; LVAT, left ventricular activation time; BI, bipolar.

Procedure characteristics are described in Table 2. The representative example of LBBAP with SDL lead is shown in Figure 2. LBBAP was achieved in 87.3% of cases (69 of 79), 86.1% were delivered via the SSPC 2 sheath, 11.4% were delivered via the SSPC 3 sheath, 2.5% were delivered via the SSPC 4 sheath. The pacing threshold was 0.8 ± 0.2 V at 0.4 ms, impedance was 792.5 ± 140.7 ohms, R wave amplitude was 14 ± 5.9 mV. The median P-V interval was 18.0 ms. The sti-LVAT was 69.0 ± 11.7 ms, QRS duration during LBBAP was 119.2 ± 11.1 ms, mean procedure time was 139.4 ± 31.5 minutes, mean fluoroscopy time was 25.5 ± 8.7 minutes.

**Table 2:** Procedural and pacing characteristics in patients with SDL-lBBAP.

Characteristics	*n* = 79
Paced QRS duration, ms	119.2 ± 11.1
Impedance, ohms	792.5 ± 140.7
R wave amplitude, mV	14.0 ± 5.9
Pacing thresholds at 0.4 ms,V	0.8 ± 0.2
LBB^1^ potential, *n* (%)	52 (65.8%)
LBBAP^2^ capture, *n* (%)	69 (87.3%)
Stimulus to LVAT^3^, ms	69.0 ± 11.7
P-V interval, ms	18.0 [14.0, 20.0]
Fluoroscopy time, min	25.5 ± 8.7
Operation time (skin to skin), min	139.4 ± 31.5
Complication	
Septal perforation, *n* (%)	6 (7.6%)
SSPC sheaths	
SSPC 2, *n* (%)	68 (86.1%)
SSPC 3, *n* (%)	9 (11.4%)
SSPC4, *n* (%)	2 (2.5%)

1 LBB, left bundle branch.

2 LBBAP, left bundle branch area pacing.

3 LVAT, left ventricular activation time.

**Figure 3:**
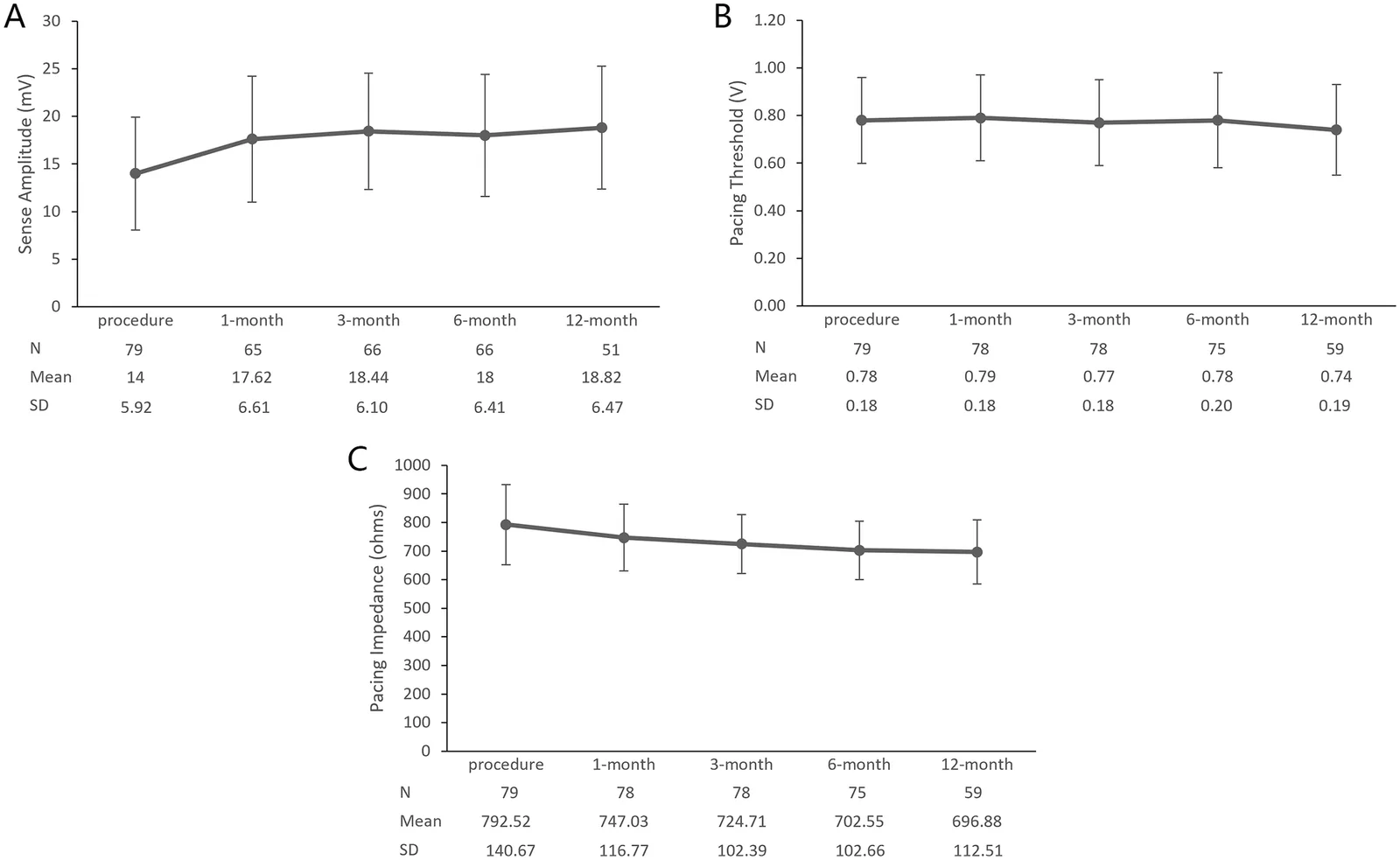
Electrical performance parameters following lBBAP with stylet-driven leads. **(A)** Pacing impedance. **(B)** R-wave sensing amplitude. **(C)** Pacing capture threshold measured intraoperatively and at 1-, 3-, 6-, and 12-months post-implantation.

**Figure 4:**
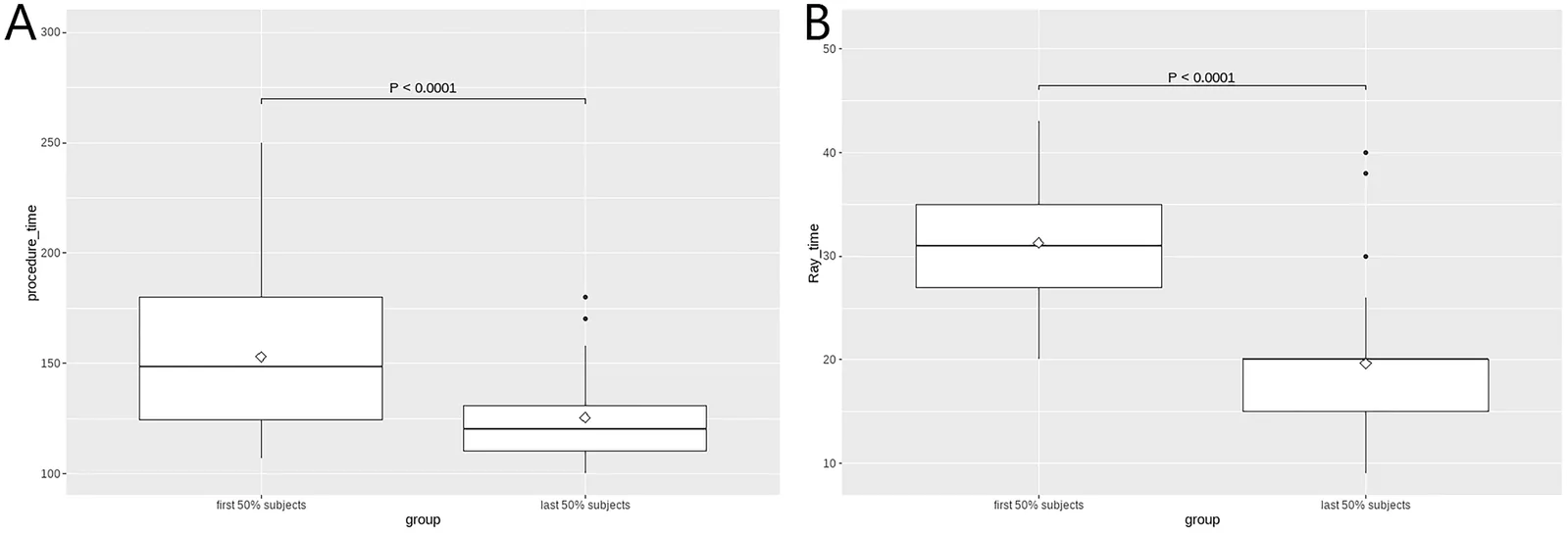
Comparison of procedural time and fluoroscopy time between the early and late halves of patients. **(A)** Procedural time between the early and late halves of patients. **(B)** Fluoroscopy time be-tween the early and late halves of patients.

Intraoperative ventricular septal perforation occurred in six patients (7.6%), with no subsequent ventricular septal defects. The R wave amplitude, pacing threshold and impedance remained stable at 1, 3, 6, and 12 months (Figure 3), with no cases of perforation or pericardial tamponade during the 12-month follow-up. Our analysis showed that both procedural time and fluoroscopy exposure time were lower in the latter 39 patients, declining markedly as operator experience accumulated (Figure 4). This finding suggests a short learning curve for the procedure. During the 12-month follow-up, no complications such as lead dislodgement, cardiac tamponade, or pocket hematoma occurred.

## 4 Discussion

While LLLs have traditionally been used, SDLs offer distinct advantages, including greater stiffness for effective septal penetration and the capability for monitoring electrocardiographic morphology continuously. A prior study (1) demonstrated that left bundle branch area pacing with SDL achieved a 94% procedural success rate at the 12-month follow-up, accompanied by low lead-related complications and stable pacing thresholds. Several clinical studies (5, 7, 8) have also confirmed the feasibility and safety of LBBAP using SDLs. However, with the stylet inserted, the SDL becomes stiffer than the LLL and may be more prone to perforating the septum when targeting the left-sided subendocardial septal area.

Optimal manifestation presents as a positive deflection in lead II and a negative deflection in lead III. Slightly higher both leads positive or lower septal positioning both negative remains acceptable and can still achieve left bundle or fascicular capture. In our clinical practice, without sheath preshaping, most cases present negative QRS waves in leads II and III, which is characteristic of left posterior fascicular capture.

Delivery sheaths vary by cardiac anatomy. The 40-cm Boston Scientific SSPC sheath comes in four configurations: Type 1 (C-curve) for His bundle pacing; Type 2 (universal) for right ventricular septal pacing; Type 3 (large curve) for enlarged right atrium and ventricular septum; Type 4 for right venous access implantation.

All septal perforations identified in our study were intraoperative micro-perforations without acute cardiac tamponade or hemodynamic compromise. Perforation was suspected based on an abrupt drop in pacing impedance during lead screwing, and confirmed under fluoroscopy. Once recognized, we immediately retracted the lead slightly, and selected an adjacent mid-septal region for lead repositioning. No patients required emergency pericardiocentesis or surgical intervention. And no procedure-related adverse events, including delayed pericardial effusion, were observed during the 12-month follow-up.

SDLs offer distinct advantages, including greater stiffness for septal penetration and the ability to monitor electrocardiographic morphology continuously. During the initial operations, septal perforation by the lead occurred easily, but no cases of cardiac tamponade or other complications were observed.

The acute success rate of LBBAP in our study was 87.3%, which was slightly lower than the 94% reported by De Pooter et al (1). Several factors may contribute to this difference. First, our study represents the initial clinical experience of the operators with the SDL-SSPC system, and the learning curve may have contributed to longer procedure duration and lower acute success rates. Second, the mechanical characteristics and implantation workflow of this newly introduced delivery system may require additional operator experience. Besides, when stable LVSP with acceptable pacing parameters was achieved, the procedure was considered successful implantation with respect to the clinical objective of providing physiological ventricular activation.

This study demonstrates that LBBAP performed with SDLs is both effective and safe, achieving high procedural success rates with favorable and stable electrical performance. However, this study involved inexperienced operators, several procedural metrics, including procedure duration and fluoroscopy time were prolonged compared to those reported in previous studies. In contrast, for experienced operators, both shorter procedure and fluoroscopy times, as well as a higher LBBP capture rate, may be anticipated. This technique simplifies LBBAP for beginners and shortens the learning curve. Our center's experience with SDLs for LBBAP: (1) Elderly, low-weight female patients, fragile myocardial tissue constitutes the main risk factor for intraoperative interventricular septal perforation. Notably, these perforations rarely result in severe complications, including pericardial effusion and cardiac tamponade. (2) Lead impedance and V1 lead QRS morphology should be closely monitored throughout the procedure, which is essential to prevent retraction of the electrode helix during the procedure. (3) During further fine adjustment after the lead is positioned, impedance and pacing morphology must be closely monitored. If fine adjustments are needed, use the torque wrench attached to the lead. Avoid directly rotating the lead body counterclockwise during fine adjustments whenever possible—this is critical for preventing septal perforation. (4) If perforation occurs, the lead should be repositioned and screwed into an adjacent site, avoiding the original perforation point. (5) If repeated attempts encounter significant resistance during lead rotation, withdraw the lead and remove it from the body to check for tissue entanglement. Clean the lead thoroughly and replace it with a new one if necessary.

There are several limitations to the present study. The cohort size receiving this implantation methodology was relatively small. Larger procedural volumes and greater operator experience are required to validate this approach. We use SSPC2 for LBBAP, but SSPC3 may be more appropriate in patients, especially in severely dilated cardiac chambers. The position tends to be inferior and can be raised by pre-shaping. We did not perform separate outcome comparisons among S-LBBAP, NS-LBBAP and LVSP. Future larger-scale studies should stratify these subgroups to investigate potential long-term differences. Prospective multicenter studies with extended follow-up are warranted to verify these findings and evaluate long-term durability.

## 5 Conclusions

Standard stylet-driven active fixation leads enable effective and safe left bundle branch area pacing with favorable acute and short-term electrical performance. SDLs possess prominent advantages: superior rigidity facilitates septal penetration, enables continuous monitoring of ECG morphology, and entails a relatively short learning curve.

## Data Availability

The raw data supporting the conclusions of this article will be made available by the authors, without undue reservation.
